# Supplementary material for: Comparing LigandMPNN and Directed Evolution for Altering the Effector-Binding Site in the RamR Transcription Factor
Source: bioRxiv. 2025 Jul 11:2025.07.10.663684. Preprint. [Version 1] doi: 10.1101/2025.07.10.663684 (PMC12265539; doi:10.1101/2025.07.10.663684)
Supplement: 12 [file NIHPP2025.07.10.663684v1-supplement-12.pdf]

bioRxiv preprint doi: https://doi.org/10.1101/2025.07.10.663684; this version posted July 11, 2025. The copyright holder for this preprint (which was not certified by peer review) is the author/funder, who has granted bioRxiv a license to display the preprint in perpetuity. It is made available under aCC-BY 4.0 International license.

Supplementary information

bioRxiv preprint doi: https://doi.org/10.1101/2025.07.10.663684; this version posted July 11, 2025. The copyright holder for this preprint (which was not certified by peer review) is the author/funder, who has granted bioRxiv a license to display the preprint in perpetuity. It is made available under aCC-BY 4.0 International license.

>Expression vector

ATGTATACCTCCTTATTAAATCAAACCCTATGAGTGAGTAAGCACTCATTATAGAAAGCAAAGATACGCCG
TCAAGGTACTTTTCAAAGGTCGACAGCCCATACAATCGATAGATTGGGACCAAAACGAAAAAAGGGGA
GCGGTTTCCCGCTCCCCTCTTTTCTGGAATTTGGTACCGAGATTGTATATGTACTTCATCTTCATTACCTCG
TATCATTGTACACCTGCCGAAAATATATTTTTCAAAAGTATCGTTTACCGCTAGCTCAGTCCTAGGTACAAT
GAGCACAGTGGCAGCCCCATAGGGTGGTGTGTACCACCCCTGATGAGTCCAAAAGGACGAAATGGGG
CCTCTACAAATAATTTTGTTTAACGGAACCACGTATCAGAAGGAGGTTAGTATATGGTAGCCCGCCCCAA
GTCGGAAGATAAGAAACAGGCTTTGTTAGAAGCGGCAACACAAGCGATTGCGCAGTCTGGAATTGCC
GCATCGACCGCTGTTATCGCACGCAATGCTGGCGTAGCTGAAGGCACTCTGTTCCGATATTTTGCCACGA
AAGATGAACTGATCAATACGTTATACCTCCATCTGAAACAAGATTTATGCCAGAGCATGATCATGGAGCT
GGACCGATCAATTACGGACGCTAAAATGATGACACGTTTCATCTGGAACTCTTACATCAGTTGGGGTCTG
AACCACCCGGCGCGCCACAGAGCCATCCGCCAGCTGGCCGTAAGCGAAAAGCTGACTAAAGAAACAG
AACAGCGGGCTGACGATATGTTTCCAGAGCTGCGTGATTTGTGTCATCGCAGTGTGTTAATGGTCTTCAT
GAGCGACGAATATCGGGCGTTCGGTGATGGCCTTTTTTTAGCACTTGCAGAAACTACGATGGATTTCGC
TGCGCGGGATCCGGCCCGCGCAGGTGAATATATTGCATTAGGTTTTGAAGCAATGTGGCGGGCTCTTAC
TCGGGAAGAACAGTAATAATCACTTTCAGCCAAAAAACTTAAGACCGCCGGTCTTGTCCACTACCTTGC
AGTAATGCGGTGGACAGGATCGGCGGTTTTCTTTTCTCTTCTCAAAGGCTAGGTGGAGGCTCAGTGATG
ATAAGTCTGCGATGGTGGATGCATGTGTCATGGTCATAGCTGTTTCCTGTGTGAAATTGTTATCCGCTCAG
AGGGCACAATCCTATTCCGCGCTATCCGACAATCTCCAAGACATTAGGTGGAGTTCAGTTCGGCGAGCG
GAAATGGCTTACGAACGGGGCGGAGATTTCCTGGAAGATGCCAGGAAGATACTTAACAGGGAAGTGA
GAGGGCCGCGGCAAAGCCGTTTTTCCATAGGCTCCGCCCCCCTGACAAGCATCACGAAATCTGACGCT
CAAATCAGTGGTGGCGAAACCCGACAGGACTATAAAGATACCAGGCGTTTCCCCCTGGCGGCTCCCTC
GTGCGCTCTCCTGTTCCTGCCTTTCGGTTTACCGGTGTCATTCCGCTGTTATGGCCGCGTTTGTCTCATTC
CACGCCTGACACTCAGTTCCGGGTAGGCAGTTCGCTCCAAGCTGGACTGTATGCACGAACCCCCCGTTC
AGTCCGACCGCTGCGCCTTATCCGGTAACTATCGTCTTGAGTCCAACCCGGAAAGACATGCAAAAGCAC
CACTGGCAGCAGCCACTGGTAATTGATTTAGAGGAGTTAGTCTTGAAGTCATGCGCCGGTTAAGGCTAA
ACTGAAAGGACAAGTTTTGGTGACTGCGCTCCTCCAAGCCAGTTACCTCGGTTCAAAGAGTTGGTAGC
TCAGAGAACCTTCGAAAAACCGCCCTGCAAGGCGGTTTTTTCGTTTTCAGAGCAAGAGATTACGCGCA
GACCAAAACGATCTCAAGAAGATCATCTTATTAAGTCTGACGCTCTATTCAACAAAGCCGCCGTCCATGG
GTAGGGGGCTTCAAATCGTCCGCTCTGCCAGTGTTACAACCAATTAACAAATTCTGATTAGAAAAACTCA
TCGAGCATCAAATGAAACTGCAATTTATTCATATCAGGATTATCAATACCATATTTTTGAAAAAGCCGTTTC
TGTAATGAAGGAGAAAACTCACCGAGGCAGTTCCATAGGATGGCAAGATCCTGGTATCGGTCTGCGATT
CCGACTCGTCCAACATCAATACAACCTATTAATTTCCCCTCGTCAAAAATAAGGTTATCAAGTGAGAAATC
ACCATGAGTGACGACTGAATCCGGTGAGAATGGCAAAAGCTTATGCATTTCTTTCCAGACTTGTTCAAC
AGGCCAGCCATTACGCTCGTCATCAAAATCACTCGCATCAACCAAACCGTTATTCATTCGTGATTGCGCCT
GAGCGAGACGAAATACGCGATCGCTGTTAAAAGGACAATTACAAACAGGAATCGAATGCAACCGGCGC
AGGAACACTGCCAGCGCATCAACAATATTTTCACCTGAATCAGGATATTCTTCTAATACCTGGAATGCTGT
TTTCCCGGGGATCGCAGTGGTGAGTAACCATGCATCATCAGGAGTACGGATAAAATGCTTGATGGTCGG
AAGAGGCATAAATTCCGTCAGCCAGTTTAGTCTGACCATCTCATCTGTAACATCATTGGCAACGCTACCTT
TGCCATGTTTCAGAAACAACTCTGGCGCATCGGGCTTCCCATACAATCGATAGATTGTCGCACCTGATTG
CCCGACATTATCGCGAGCCCATTTATACCCATATAAATCAGCATCCATGTTGGAATTTAATCGCGGCCTCG
AGCAAGACGTTTCCCGTTGAATATGGCTCATAACACCCCTTGTATTACTGTTTATGTAAGCAGACAGTTTT
ATTGTTCATGATGATATATTTTTATCTTGTGCAATGTAACATCAGAGATTTTGAGACACAACGTGGCTTTCC

bioRxiv preprint doi: https://doi.org/10.1101/2025.07.10.663684; this version posted July 11, 2025. The copyright holder for this preprint (which was not certified by peer review) is the author/funder, who has granted bioRxiv a license to display the preprint in perpetuity. It is made available under aCC-BY 4.0 International license.

CCCGCCGCTCTAGAACTAGTGGATCCAAATAAAACGAAAGGCTCAGTCGAAAGACTGGGCCTTTCGTTT
TATCTGTTGTTTGTCGCATTATACGAGACGTCCAGGTTGGGATACCTGAAACAAAACCCATCGTACGGCC
AAGGAAGTCTCCAATAACTGTGATCCACCACAAGCGCCAGGGTTTTCCCAGTCACGACGTTGTAAAACG
ACGGCCAGTCATGCATAATCCGCACGCATCTGGAATAAGGAAGTGCCATTCCGCCTGACCTGGACCAAA
ACGAAAAAAGGGGAGCGGTTTCCCGCTCCCCTCTTTTCTGGAATTTGGTACCGAGGAATGAAGCAGGA
TTATTATTTGTATAGTTCATCCATGCCATGTGTAATCCCAGCAGCTGTTACAAACTCAAGAAGGACCATGT
GGTCTCTCTTTTCGTTGGGATCTTTCGAAAGGGCAGATTGTGTGGACAGGTAATGGTTGTCTGGTAAAA
GGACAGGGCCATCGCCAATTGGAGTATTTTGTTGATAATGGTCTGCTAGTTGAACGCTTCCATCTTCAAT
GTTGTGTCTAATTTTGAAGTTAACTTTGATTCCATTCTTTTGTTTGTCTGCCATGATGTATACATTGTGTGA
GTTATAGTTGTATTCCAATTTGTGTCCAAGAATGTTTCCATCTTCTTTAAAATCAATACCTTTTAACTCGATT
CTATTAACAAGGGTATCACCTTCAAATTTGACTTCAGCACGTGTCTTGTAGTTCCCGTCATCTTTGAAAAA
TATAGTTCTTTCCTGTACATAACCTTCGGGCATGGCACTCTTGAAAAAGTCATGCTGTTTCATATGATCTG
GGTATCTCGCAAAGCATTGAAGACCATACGCGAAAGTAGTGACAAGTGTTGGCCATGGAACAGGTAGT
TTTCCAGTAGTGCAAATAAATTTAAGGGTAAGTTTTCCGTATGTTGCATCACCTTCACCCTCTCCACTGAC
AGAAAATTTGTGCCCATTAACATCACCATCTAATTCAACAAGAATTGGGACAACTCCAGTGAAAAGTTCT
TCTCCTTTACTCAT

RamR-responsive promoter
Terminator
P250 constitutive promoter
RamR
P15a
Kanamycin resistance
GFP

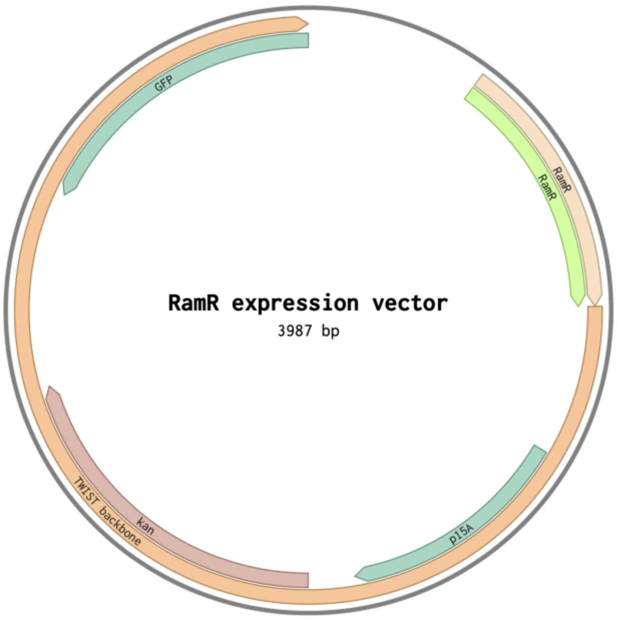

bioRxiv preprint doi: https://doi.org/10.1101/2025.07.10.663684; this version posted July 11, 2025. The copyright holder for this preprint (which was not certified by peer review) is the author/funder, who has granted bioRxiv a license to display the preprint in perpetuity. It is made available under aCC-BY 4.0 International license.

Supplementary table 1. Expression plasmid sequence. Components of the plasmid are highlighted, and a map of the plasmid is provided at the bottom of the table.

Protein variant	Protein sequence
Wild-type RamR	MVARPKSEDKKQALLEAATQAIAQSGIAASTAVIARNAGVAEGTLFRYFATKDELI<br>NTLYLHLKQDLCQSMIMELDRSITDAKMMTRFIWNSYISWGLNHPARHRAIRQL<br>AVSEKLTKETEQRADDMFPELRDLCHRSVLMVFMSDEYRAFGDGLFLALAETTM<br>DFAARDPARAGEYIALGFEAMWRALTREEQ

Supplementary table 2. Wild-type RamR protein sequence.

Protein variant	Protein sequence
Evolved Round 4	MVARPKSEDKKQALLEAATQAIAQSGIAASTAVIARNAGVAEGTLFRYFATKDELI<br>NTLYLHLTQDWCQSIIMELDRSITDAKMMTRFLWNSWISWGLNHPARHRAIRQ<br>LAVSEKLTKETEQRADDMFPELRDHLHRNVLMVFMSDEYRAFGDGLFLALAETT<br>MDFAARDPARAGEYIALGFEAMWRALAREEQ
LigandMPNN: AlphaFold-DiffDock	MVARPKSEDKKQALLEAATQAIAQSGIAASTAVIARNAGVAEGTLFRYFATKDELI<br>NTLYLHLMQDCCQSMIMELDRSITDAKMMFRFFWNSYISWGLNHPARHRAIRQ<br>LAVSEKLTKETLQRAEDMFPELRDLMHRRVLMVFMSDEYRAFGAGLFLALAETT<br>MDFAARDPARAGEYIALGFEAMWRALTREEQ
LigandMPNN: RFAA	MVARPKSEDKKQALLEAATQAIAQSGIAASTAVIARNAGVAEGTLFRYFATKDELI<br>NTLYLHLVQDLCQSIIMELDRSITDAKMMARFIWNSFISWGLNHPARHRAIRQLA<br>VSEKLTKETLQRAGDMFPELRDLIHRSVLMVFMSDEYRAFGIGLFLALAETTMDF<br>AARDPARAGEYIALGFEAMWRALTREEQ

Supplementary table 3. GLAU protein variants.

Protein variant	Protein sequence
Evolved Round 4	MVARPKSEDKKQALLEAATQAIAQSGTAASTAVIARNAGVAEGTLFRYFATKDELI<br>NTLYLHLFQDWCQSSIMELDRSITDAKMMTRFLWNSIISWGLNHPARHRAIRQL

bioRxiv preprint doi: https://doi.org/10.1101/2025.07.10.663684; this version posted July 11, 2025. The copyright holder for this preprint (which was not certified by peer review) is the author/funder, who has granted bioRxiv a license to display the preprint in perpetuity. It is made available under aCC-BY 4.0 International license.

	AVSEKLSKETVQRADDMFPELRDIVHREVLMVFMSDEYRAFGEGLFLALAETTM<br>DFAARDPARAGEYIALGFEAMWRALTREEQ
LigandMPNN:<br>AlphaFold-<br>DiffDock	MVARPKSEDKKQALLEAATQAIAQSGIAASTAVIARNAGVAEGTLFRYFATKDELI<br>NTLYLHLVQDLCQSMIMELDRSITDAKMMARFIWNSYISWGLNHPARHRAIRQL<br>AVSEKLTKETLQRAEDMFPELRDLEHRRVLMVFMSDEYRAFGVGLFLALAETTM<br>DFAARDPARAGEYIALGFEAMWRALTREEQ
LigandMPNN:<br>RFAA	MVARPKSEDKKQALLEAATQAIAQSGIAASTAVIARNAGVAEGTLFRYFATKDELI<br>NTLYLHLGQDLCQSIIMELDRSITDAKMMARFVWNSFISWGLNHPARHRAIRQL<br>AVSEKLTKETLQRWGDMFPELRDLMHRTVLMVFMSDEYRAFGVGLFLALAETT<br>MDFAARDPARAGEYIALGFEAMWRALTREEQ

Supplementary table 4. THP protein variants.

Protein variant	Protein sequence
Evolved Round 4	MVARPKSEDKKQALLEAATQAIAQSGIAASTAVIARNAGVAEGTLFRYFATKDELI<br>NTLYLHLTHDMCQSLIMELDRSITDAKMMTRFIWNSYISWGLNHPARHRAIRQL<br>AVSEKLTKETRQRARDMFPELRDLCYRSLLMVFMSDEYRAFGDGLFMALAETTM<br>DFAARDPARAGEYIALGFEAMWRALTREEQ
LigandMPNN:<br>AlphaFold-<br>DiffDock	MVARPKSEDKKQALLEAATQAIAQSGIAASTAVIARNAGVAEGTLFRYFATKDELI<br>NTLYLHLMQDVCQSMIMELDRSITDAKMMARFIWNSYISWGLNHPARHRAIRQ<br>LAVSEKLTKETLQRAEDMFPELRDLNHRLVLMVFMSDEYRAFGRGLFLALAETTM<br>DFAARDPARAGEYIALGFEAMWRALTREEQ
LigandMPNN:<br>RFAA	MVARPKSEDKKQALLEAATQAIAQSGIAASTAVIARNAGVAEGTLFRYFATKDELI<br>NTLYLHLLQDWCQSVIMELDRSITDAKMMARFVWNSFISWGLNHPARHRAIRQ<br>LAVSEKLTKETLQRAEDMFPELRDLRHRLVLMVFMSDEYRAFGVGLFLALAETTM<br>DFAARDPARAGEYIALGFEAMWRALTREEQ

Supplementary table 5. NOS protein variants.

Protein variant	Protein sequence

bioRxiv preprint doi: https://doi.org/10.1101/2025.07.10.663684; this version posted July 11, 2025. The copyright holder for this preprint (which was not certified by peer review) is the author/funder, who has granted bioRxiv a license to display the preprint in perpetuity. It is made available under aCC-BY 4.0 International license.

Evolved Round 4	MVARPKSEDKKQALLEAATQAIAQSGIAASTAVIARNAGVAEGTLFRYFATKDELI NTLYLHLRQDLCQSLIMELDRSITDAKMMMRFIWNSGISWGLNHPARHRAIRQL AVSEKLTKETHQRDLDMFPELRDILHRRVLMVFMSDEYRAFGDGLFLALAETTM DFAARDPARAGEYIALGFEAMWRALTREEQ
LigandMPNN: AlphaFold-DiffDock	MVARPKSEDKKQALLEAATQAIAQSGIAASTAVIARNAGVAEGTLFRYFATKDELI NTLYLHLMQDLCQSMIMELDRSITDAKMMARFFWNSFISWGLNHPARHRAIRQ LAVSEKLTKETLQRAEDMFPELRDLLHRRVLMVFMSDEYRAFGAGLFLALAETTM DFAARDPARAGEYIALGFEAMWRALTREEQ
LigandMPNN: RFAA	MVARPKSEDKKQALLEAATQAIAQSGIAASTAVIARNAGVAEGTLFRYFATKDELI NTLYLHLWQDLCQSIIMELDRSITDAKMMARFVWNSFISWGLNHPARHRAIRQL AVSEKLTKETLQRAGDMFPELRDLMHRTVLMVFMSDEYRAFGIGLFLALAETTM DFAARDPARAGEYIALGFEAMWRALTREEQ

Supplementary table 6. PAP protein variants.

**Protein variant**	**Protein sequence**
Evolved Round 4	MVARPKSEDKKQALLEAATQAIAQSGIAASTAVIARNAGVAEGTLFRYFATKDELI NTLYLHLYQDHCQSLIMELDRSITDAKMMIRFTWNSYISWGLNHPARHRAIRQLA VSEKLTKETKQRIEDMFPELRDILHRSVLMVFMSDEYSAFGKGLFYALAETTMDFA ARDPARAGEYIALGFEAMWRALTREEQ
LigandMPNN: AlphaFold-DiffDock	MVARPKSEDKKQALLEAATQAIAQSGIAASTAVIARNAGVAEGTLFRYFATKDELI NTLYLHLIQDLCQSMIMELDRSITDAKMMARFIWNSFISWGLNHPARHRAIRQL AVSEKLTKETLQRAEDMFPELRDLEHRLVLMVFMSDEYRAFGRGLFLALAETTM DFAARDPARAGEYIALGFEAMWRALTREEQ

Supplementary table 7. ROTU protein variants.

**Amino Acid**	**Round 4 Directed Evolution variants**	**LigandMPNN design variants**
R	4	6

bioRxiv preprint doi: https://doi.org/10.1101/2025.07.10.663684; this version posted July 11, 2025. The copyright holder for this preprint (which was not certified by peer review) is the author/funder, who has granted bioRxiv a license to display the preprint in perpetuity. It is made available under aCC-BY 4.0 International license.

H	3	0
K	2	0
D	1	0
E	2	7
S	2	0
T	4	2
N	1	1
Q	0	0
C	0	1
G	1	4
P	0	0
A	0	10
V	2	10
I	7	7
L	9	14
M	3	6
F	1	10
Y	2	0
W	3	3

Supplementary table 8. Amino acid distribution of designed residues.
